# Supplementary material for: Selection and Characterization of Monoclonal Antibodies Targeting Middle East Respiratory Syndrome Coronavirus through a Human Synthetic Fab Phage Display Library Panning
Source: Antibodies (Basel). 2019 Jul 31;8(3):42. doi: 10.3390/antib8030042 (PMC6783954; doi:10.3390/antib8030042)
Supplement: Supplementary file 1 [file antibodies-08-00042-s001.pdf]

MDPI

Author statements

Please insert the relevant text under the subheadings below. A completed form must be signed by all authors. Please note that we only accept hand-signed signatures. Please upload the signed copy with your submission, otherwise we will not process your manuscript further.

Manuscript title: Selection and Characterization of Monoclonal Antibodies Targeting MERS-CoV through a Human Synthetic Fab Phage Display Library Panning

Corresponding author: Dae Young Kim

Article type: Original Research Article

Does your manuscript have an ethic approval number? No ▣ Yes ☐ If yes, enter number here:

Authors' contributions

We follow ICMJE recommendation of authorship, which is based on the following 4 criteria:

1. Substantial contributions to the conception or design of the work; or the acquisition, analysis, or interpretation of data for the work; AND
2. Drafting the work or revising it critically for important intellectual content; AND
3. Final approval of the version to be published; AND
4. Agreement to be accountable for all aspects of the work in ensuring that questions related to the accuracy or integrity of any part of the work are appropriately investigated and resolved.

All those designated as authors should meet all four criteria for authorship, and all who meet the four criteria should be identified as authors. Those who do not meet all four criteria should be acknowledged. Please insert here the contribution each author made to the manuscript—e.g., literature search, figures, study design, data collection, data analysis, data interpretation, writing etc. If all authors contributed equally, please state this. The information provided here must match the contributors' statement in the manuscript.

Author Contributions: D.Y.K., H.L., K.P., and S.S.K. conceived and designed the experiments. Y.K., S.P., J.-H.L., M.K.S., H.W., H.K., and J.-M.L. performed the experiments. S.H.L., B.J.K., Y.-S.P., S.-Y.C., D.H.S., and J.-Y.L. analyzed the data. D.Y.K. wrote the article.

Conflicts of interest

Please insert here: 1. authors' conflicts of interest; 2. sources of support for the work, including sponsor names along with explanations of the role of those sources if any in study design; collection, analysis, and interpretation of data; writing of the report; 3. the decision to submit the report for publication; or a statement declaring that the supporting source had no such involvement, etc.

Conflicts of Interest: Y.K., K.P., H.W., H.K., H.L., S.S.K., J.-Y.L., and D.Y.K. are inventors on Korea patent number 10-1969696 (registration date April 10, 2019). S.H.L., J.-M.L, and Y.-S.P. are employees of Plexense Inc. The authors declare no other conflicts of interest.

Funding: This research was supported by grants from Korea Center for Disease Control (KCDC) (2016-ER4806-00 & 2016-NG47001-00), and also by grants from Chungcheongbuk-do Value Creation Project (Plexense) and MSIT New Drug Development Center R&D Supporting Project (Human synthetic antibody library). The two KCDC grants contributed phage panning, antibody production and characterization, and neutralization assay. The grants from Chungcheongbuk-do Value Creation Project (Plexense) and MSIT New Drug Development Center R&D Supporting Project (Human synthetic antibody library) contributed ACCEL ELISA work and human synthetic Fab phage display library, respectively.

I agree with: the plan to submit to *Antibodies;* the contents of the manuscript; to being listed as an author; and to the conflicts of interest statement as summarised. I have had access to all the data in the study (for original research articles) and accept responsibility for its validity.

Title and name	Highest degree	Signature	Date
Title and name: Dr. Dae Young Kim	Highest degree: Ph.D.	Signature:	Date: 2019.08.08.
Title and name: Ms. Yoonji Kim	Highest degree: M.Sc.	Signature:	Date: 2019.08.08.
Title and name: Dr. Hansaem Lee	Highest degree: Ph.D.	Signature:	Date: 2019.08.08.
Title and name: Dr. Keunwan Park	Highest degree: Ph.D.	Signature:	Date: 2019.08.08.
Title and name: Ms. Sora Park	Highest degree: M.Sc.	Signature:	Date: 2019.08.08.
Title and name: Ms. Ju-Hyeon Lim	Highest degree: M.Sc.	Signature:	Date: 2019.08.08.
Title and name: Ms. Min Kyung So	Highest degree: M.Sc.	Signature:	Date: 2019.08.08.
Title and name: ~~Ms.~~ Dr. Hye-Min Woo	Highest degree: ~~M.Sc.~~ Ph.D.	Signature:	Date: 2019.08.08.
Title and name: Ms. Hyemin Ko	Highest degree: M.Sc.	Signature:	Date: 2019.08.08.
Title and name: Dr. Jeong-Min Lee	Highest degree: Ph.D.	Signature:	Date: 2019.08.08.
Title and name: Dr. Sun Hee Lim	Highest degree: Ph.D.	Signature:	Date: 2019.08.08.
Title and name: Dr. Byoung Joon Ko	Highest degree: Ph.D.	Signature:	Date: 2019.08.08.
Title and name: Dr. Yeon-Su Park	Highest degree: Ph.D.	Signature:	Date: 2019.08.08.
Title and name: Dr. So Young Choi	Highest degree: Ph.D.	Signature:	Date: 2019.08.08.
Title and name: Mr. Du Hyun Song	Highest degree: M.Sc.	Signature:	Date: 2019.08.08.
Title and name: Dr. Joo–Yeon Lee	Highest degree: Ph.D.	Signature:	Date: 2019.08.08.
Title and name: Dr. Sung Soon Kim	Highest degree: Ph.D.	Signature:	Date: 2019.08.08.

Corresponding author declaration

I Dae Young Kim, the corresponding author of this manuscript, certify that the contributors' and conflicts of interest statements included in this paper are correct and have been approved by all co-authors.
